# Cryogenic preservation of *Dirofilaria immitis* microfilariae, reactivation and completion of the life-cycle in the mosquito and vertebrate hosts

**DOI:** 10.1186/s13071-021-04839-7

**Published:** 2021-07-16

**Authors:** Erich W. Zinser, Tom L. McTier, Nicole S. Kernell, Debra J. Woods

**Affiliations:** Zoetis, Veterinary Medicine Research and Development, 333 Portage St, Kalamazoo, MI 49007 USA

**Keywords:** Cryopreservation, Microfilariae, *Dirofilaria immitis*

## Abstract

**Background:**

The cryopreservation of filarial nematodes has been studied for nearly 70 years. Largely, these studies examined the effectiveness of cryopreservation methods by using the post-thaw survival of microfilariae (mf) and the development to third-stage larvae (L3s) following inoculation into a competent insect vector. Only one study reported complete reestablishment of a filarial nematode (*Brugia malayi*) life-cycle in a competent vertebrate host from cryopreserved stock. Expanding on this previous research, a cryopreservation method was developed to cryopreserve the mf of the dog heartworm, *Dirofilaria immitis*.

**Methods:**

A combination of cryoprotectants, dimethyl sulfoxide (DMSO) and polyvinyl pyrolidone (PVP) at 6% and 4 mM, respectively, provided acceptable post-thaw survival of mf that developed into L3s in *Aedes aegypti*. L3s developed from cryopreserved and freshly collected mf in mosquitoes were inoculated into ferrets and dogs and were assessed after a sufficient duration post-inoculation for development into adult heartworms.

**Results:**

Fewer adult heartworms derived from cryopreserved stocks of mf were recovered from ferrets compared to adult heartworms derived from freshly collected mf, and the former were smaller by weight and length. The onset of patency (circulating mf) occurred at similar post-inoculation time points and at similar mf densities in dogs infected with L3s sourced from cryopreserved stocks or freshly collected mf. Adults derived from cryopreserved mf have survived and produced viable mf for more than 3 years in dogs. Approximately 60% of inoculated L3s were recovered as adults from dogs at 2 and 3.5 years post-inoculation.

**Conclusions:**

The results from these direct comparisons demonstrate that cryopreserved mf can develop into L3s in vector mosquitoes and that these L3s are infective to both dogs and ferrets, where they undergo normal development into adult worms. These worms are able to mate and produce viable mf and complete the heartworm lifecycle in dog.

**Graphical Abstract:**

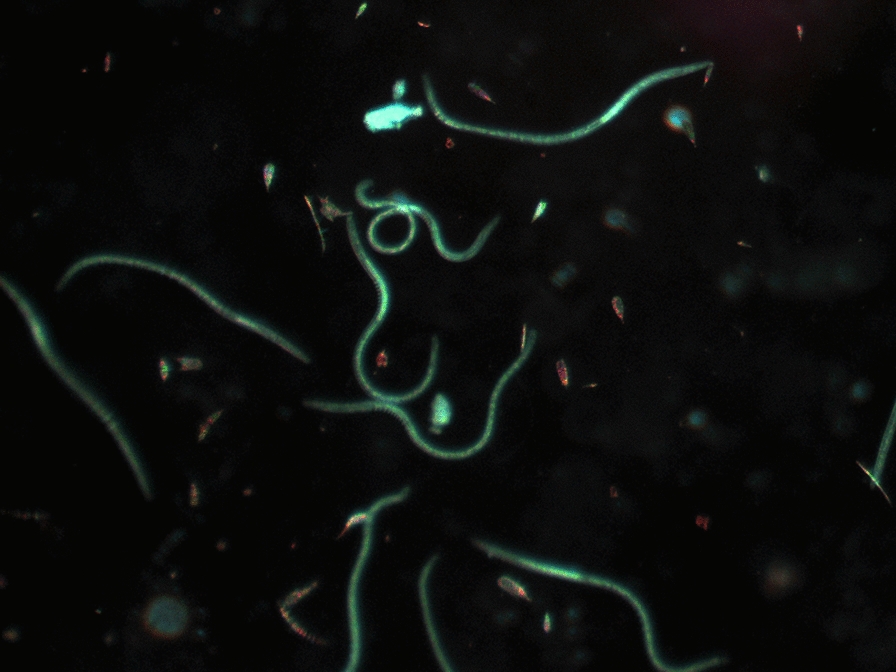

## Background

*Dirofilaria immitis* is a parasitic filarial nematode, primarily of canids [1]. Macrocyclic lactones (MLs) are used in a number of commercial products to prevent heartworm disease in dogs and other species (e.g. cats and ferrets). Reports of loss of efficacy of these products have been reported [2–4], and heartworms showing ML-resistant phenotypes and genotypes have been isolated from the field [5–7]. ML-resistant and susceptible populations may have characteristics important for research into the mechanisms of host avoidance and drug resistance. Heartworm isolate maintenance is resource and animal intensive, involving the use of numerous dogs to maintain specific isolates of heartworms in order to have the stages of the parasite readily available for study. The presence of adult heartworms causes progressive disease in these dogs, and the dogs must be carefully monitored and given special care to prevent the disease from becoming debilitating or even fatal. Limiting the use of animals in research is ethically proper and has been strongly encouraged and widely adopted across biomedical research organizations. The intent is to reduce the pain, suffering and distress on animals that may result from diseases induced to study disease progression, and the use of scientific procedures needed to develop novel treatments and preventatives for heartworm disease.

Cryogenic preservation (cryopreservation) is the freezing of an organism in a viable state, with the intention that the organism can be thawed and brought back to a normal state at some time in the future. A summary of filarial nematode cryopreservation literature is shown in Table 1. Nearly all of these studies assessed successful cryogenic storage based on microfilaria (mf) survival, mf motility and the development of third-stage larvae (L3s) in insect vectors following mf thawing. Microscopic analysis by Taylor [8] showed that while survival and motility may be largely unaffected, there are structural changes that arise from cryopreservation. Only Lowrie [9] has reported successful completion of the whole filarial nematode life-cycle of *Brugia malayi* following cryopreservation of mf.

**Table 1:** Summary of key literature describing the tested cryopreservation conditions and outcomes for several species of filarial nematodes

Species^a^	Experimental conditions				Summary of outcomes	Reference
	Additives	Freezing conditions				
		Rate (°C/min)	Duration (days)	Final temperature (°C)		
*Di*,* Wb*,* Lc*	None	No control	1–69	− 15 and − 70	Based on post-thaw motility, mf survival was low for *Wb*, 60–95% survival of *Di*, 38–54% survival of *Lc*	[17]
*Ll*,* Wb*,* Bm*, *Di*,* Dr*,*Da*,* Lc*	Glycerol with serum	No control	Not stated	− 79	50–80% survived and were motile, but phase contrast and ultraviolet microscopy showed morphological changes	[18]
*Di*	None	No control	660	− 68	Thawed mf successfully developed to L3s in mosquito, but with low rate of recovery compared to unfrozen control	[19]
*Oc*,* Og*,* Ov*	5% Methanol 5% DMSO 5% Ethanediol 5% Glycerol	1	Not stated	− 196	Thawed mf were successfully isolated from mammalian host following injection. Viable L3s were isolated from competent insect vectors following injection. 5% Methanol as cryoprotectant showed improved recovery rates compared to other cryoprotectants tested	[20]
*Di*,* Dr*,* Og*,* Bp*	16% HES	1.3 to > 200	Not stated	− 196	Viable L3s were isolated from insect vector after feeding. Cooling rate of 1.3 C/min showed best recovery of viable L3s. HES does not require washing after thaw and thawed blood can be fed directly to insect vector	[21]
*Di*—mf and L3	mf—5% DMSO with 20% newborn calf serum L3—as above, with or without 16% HES	1	Not stated	− 196	Motility and rate of L3 recovery was lower for thawed mf compared to fresh mf following fluid enema of mf into mosquito. L3 survival after thawing was similar to unfrozen controls, but rate of molting to L4 was very low	[22]
*Dc*,* Bm*,* Wb*	*Dc*—6% DMSO with 4 mM PVP *Bm*—9% DMSO	0.5–10	4–540	− 196	*Dc*, *Bm* and *Wb* L3 successfully developed to L3 in insect vector after feeding. Resulting *Bm* L3s injected into primate host, and life-cycle was successfully completed	[9]
*Bm*	6% DMSO with 15% newborn calf serum	0.5–1.0	6–375	− 196	Post-thaw survival of mf was 94–98%. Motility and L3 development in insect vector after feeding were similar to those of unfrozen control	[23]
*Di*,* Bm*,* Wb*	6% DMSO with 4 mM PVP	1	30	− 196	Post-thaw motility of mf, and L3 recovery from insect vector after feeding was comparable to that of unfrozen controls	[24]

*DMSO* Dimethyl sulfoxide, *HES* Hydroxyethyl starch),*L3* third-stage larval form,* mf* microfilariae,* PVP* olyvinyl pyrolidone

^a^*Di*, *Dirofilaria immitis*; *Dr*, *Dirofilaria repens*; *Dc*, *Dirofilaria corynodes*; *Da, Dirofilaria aethiops*; *Ll, Loa loa; Og*, *Onchocerca gutturosa*; *Oc*, *Onchocerca cervicalis*; *Ov*, *Onchocerca volvulus*; *Bp*, *Brugia pahangi*.; *Bm*, *Brugia malayi*; *Wb*, *Wuchereria bancrofti*; *Lc*, *Litomosoides carinii* (*now L. sigmodontis*)

Successful cryopreservation and recovery of one of the life stages of *D. immitis*, namely the mf, would reduce the number of dogs needed for isolate maintenance while allowing for the long-term conservation and future study of viable, distinct heartworm isolates. The three objectives of this study were: (i) to establish a suitable method for *D. immitis* mf cryopreservation by recovering viable mf after thawing, then collect L3s from mosquitoes that had been fed the thawed mf; (ii) to confirm the infectivity of the L3s obtained from mosquitoes in ferrets and dogs; and (iii) to determine the reproductive fitness of adult heartworms that developed from the L3s following reestablishment in dogs.

## Methods

### Cryopreservation, thawing and assessment of survival

Glycerol, dimethyl sulfoxide (DMSO) and polyvinyl pyrolidone (PVP; 40,000 MW) are commonly used cryoprotectants (summarized in Table 1). We selected these cryoprotectants for evaluation in six cryoprotectant combinations to determine successful freezing and recovery conditions for mf. Sterile 0.9% irrigation saline was used to make double-strength stock solutions of 10% DMSO, 12% DMSO, 10% glycerol/8 mM PVP, 10% DMSO/8 mM PVP and 12% DMSO/8 mM PVP. Blood containing circulating mf was collected directly into tubes containing ethylenediaminetetraacetic acid (EDTA) as an anticoagulant, diluted 50:50 in each cryoprotectant and mixed thoroughly. Aliquots of 1–1.5 ml were quickly dispensed into 2-ml cryovials; these were first placed in a Nalgene™ Cryo 1° Freezing Container (Cat. No. 5100–0001; Thermo Fisher Scientific, Waltham, MA, USA) that was then placed at − 80 °C for > 24 h before being plunged into liquid nitrogen for long-term storage. The final concentrations of cryoprotectants in the blood were 5% DMSO, 6% DMSO, 5% glycerol/4 mM PVP, 5% DMSO/4 mM PVP and 6% DMSO/4 mM PVP, respectively.

*Aedes* saline (9 g sodium chloride, 0.2 g calcium chloride, 0.2 g potassium chloride and 0.1 of sodium bicarbonate disolved in 1 l distilled water [10]) was used to maintain freshly thawed mf near physiological salinity. Samples were thawed by submerging the cryotubes into a 37 °C water bath until they were completely thawed (< 2 min). The contents of each cryotube were then poured into a 15-ml disposable centrifuge tube, diluted to 12 ml in chilled *Aedes* saline, mixed gently and then centrifuged at 2000 rpm for 5 min. The supernatant was carefully removed, and the *Aedes* saline rinse/centrifugation process was repeated two more times to completely remove the cryoprotectant. The supernatant was carefully removed after the third rinse, leaving 1–2 ml of saline and the pellet containing mf in the tube.

For* in vitro* examination of mf, the pellet was diluted with 13 ml of RPMI-1640 medium (+ 1% penicillin/streptomycin and 5% fetal bovine serum), transferred to a 30-m cell culture flask and incubated at 37 °C. Qualitative assessment of mf motility was performed at 6 h post-thaw, and motility and survival counts were performed at 3 and 5 days post-thaw. Motility was scored as fast, medium and slow by observing the entire area of the flask. Survival counts were performed on three random sections of each flask by counting live and dead worms. Averages were calculated for each cryoprotectant at each time point, and percentage survival was determined.

For mosquito inoculation, 5 ml of normal, mf-free dog blood collected in lithium heparin was added to the mf pellet; this mixture was maintained at room temperature until further dilution to 3500 mf/ml with mf-free blood prior to mosquito (*A. aegypti*—Liverpool strain) feeding. Mosquitoes were incubated for 14–16 days at 26 °C and 80% relative humidity to allow the mf to undergo development to L3s [11].

### Heartworm inoculation

L3s produced from both freshly collected (FCmf) and frozen (CPmf) mf were harvested from *A. aegypti* after 14–16 days of mosquito incubation. Adult mosquitoes were anesthetized in a freezer (2 min) and then lightly crushed with a mortar and pestle with chilled Hank’s Balanced Salt Solution (HBSS). The contents of the mortar and pestle were placed in a No. 140 mesh sieve and washed twice with chilled HBSS to remove large debris. The washings were removed, and contents of the sieve were floated in 37 °C HBSS for 3 min to allow the L3s to warm and actively migrate through the sieve and into the Petri dish below. The contents of the Petri dish were kept, and the sieve contents were washed with warm HBSS for another three to four times.

A stereo dissecting microscope and a small Pasteur pipette were used to count the L3s from the Petri dishes where they had been collected. Larvae were examined qualitatively for size and motility, and the largest and most motile larvae were added to a 40-mm Petri dish that was tilted slightly to allow the larvae to settle to one end of the dish. Fluid was removed and replaced three times to rinse the remaining debris. Excess fluid was then removed from the dish such that < 1 ml of fluid (containing larvae) remained in the dish. The fluid containing the larvae was drawn into a 1-ml tuberculin syringe, and a 22-gauge needle was then attached. The syringes were held at room temperature until inoculation. To inoculate the animals, the contents of the syringe were injected into the animal subcutaneously in the inguinal area. The syringe was rinsed several times with media and the contents re-injected to ensure that all larvae were removed from the syringe. The syringe was then rinsed in a Petri dish and examined microscopically for remaining larvae. Any remaining larvae were re-injected into the animal as described above. Using the above technique, animals were inoculated on Day 0. Each ferret received 25 *D. immitis* L3s and dogs received 40 L3s.

### Infectivity and fitness of L3s in ferrets

One of the objectives of this study was to determine the ability of *D. immitis* L3s to develop to maturity in ferrets following cryogenic preservation of the mf stage. Mf were cryogenically preserved for 4 weeks in 6% DMSO/4 mM PVP, thawed and passed through *A. aegypti* to the L3 stage prior to inoculation into the ferret as described in the previous section. Three animals were assigned to each of two treatment groups to receive L3s harvested from mosquitoes that were fed mf from two sources. Animals allotted to T01 received L3s derived from FCmf; animals allotted to T02 received L3s that were derived from CPmf that had been cryopreserved for 4 weeks. Animals were housed in triplicate, with each treatment group housed in a single cage, confounding cage assignment and treatment.

All animals were inoculated with L3s by subcutaneous injection on Day 0 and maintained until necropsy. Blood was collected for adult heartworm antigen testing on Days 5, 90, 120 and 133 post-inoculation. Animal C83 (T02) began to show abnormal urine and emesis on Day 113 that may have been related to the heartworm inoculation and progression of heartworm disease. Animal C83 and a corresponding animal from T01, animal C63, were euthanized on Day 113. All remaining study subjects were euthanized on Day 133. *Dirofilaria immitis* will not reliably proceed to patency (circulating mf) in most ferrets, so adult counts and measurements of length and weight were used to assess the fitness of FCmf and CPmf. After euthanasia, heartworms from all study subjects were collected, sexed and counted. The length of each intact worm was measured following removal from the infected animal on Days 113 and 133. Intact worms from each ferret were pooled by sex and weighed on Day 133 only.

Due to confounding of cage and treatment, the experimental unit for treatment in this study was the cage with no replication, with each animal being an observational unit. The study was not appropriately powered to define treatment differences, and statistical analysis was conducted post hoc. Assuming there was no effect of housing on heartworm recovery and treating the animal as the experimental unit, total adult worm counts from each animal, average adult worm lengths on Day 133 and average adult worm weights on Day 133 were log transformed [log_10_(mf + 1)].* P*-values comparing treatment means were generated using Student’s* t*-test on the log-transformed results, and assessed at the two-sided 10% level of significance.

### L3 infectivity and fitness in dogs

The second objective of this study was to determine the ability of *D. immitis* L3s to develop to maturity in dogs following cryogenic preservation of the mf stage and to assess the time of onset of patency and if CPmf produced similar numbers of circulating mf as FCmf. Mf were cryogenically preserved in 6% DMSO/4 mM PVP for 12 weeks, thawed and passed through *A. aegypti* to L3s prior to inoculation into dogs, as described above. Two animals were assigned to each of two treatment groups to receive L3s harvested from mosquitoes that were fed mf from the two sources. Animals allotted to T01 received L3s that were derived from FCmf, and T02 animals received L3s from CPmf. All animals were inoculated on Day 0. Blood was collected and analyzed for circulating mf using the Knott’s test [12] starting at 20 weeks post-inoculation (Day 140). A DiroCHEK® (Zoetis, Parsippany, NJ) heartworm antigen test was conducted at 27 weeks (Day 198) post-inoculation. Animals were individually housed throughout the duration of the study, and the animal was the experimental unit. Mf counts at each time point were log transformed [log_10_(mf + 1)] Student’s* t*-test was used to compare treatment means for log-transformed mf counts for weeks 26, 28, 30 and 36 post-inoculation at the two-sided 10% level of significance.

## Results

### Assessment of mf survival following cryopreservation

Qualitative assessment of motility was similar across all cryoprotectants at each time point. Mf showed higher survival in cryoprotectants that contained DMSO and PVP (Table 2), indicating that both are important components to maximize the survival of frozen mf. Experimental cryoprotectant combinations that contained DMSO showed increased mf survival of about 1.8- to 2.5-fold higher than glycerol at 3 and 5 days post-thaw, respectively. PVP also improved the survival of mf, with the glycerol/DMSO groups all showing higher survival than glycerol or DMSO alone. While there was no appreciable difference between 5 and 6% DMSO with 4 mM PVP, 5% DMSO alone showed improved survival over 6% DMSO alone at 3 and 5 days post-thaw.

**Table 2:** Percentage survival and qualitative motility observed of *Dirofilaria immitis* microfilariae at 6 h and 3 and 5 days after thawing from a cryopreserved state of − 80 °C for > 30 days

Day post-thaw	Survival parameter	Cryoprotectant/cryoprotectant combination					
		5% Glycerol	5% DMSO	6% DMSO	5% Glycerol/PVP^a^	5% DMSO/PVP^a^	6% DMSO/PVP^a^
Day 0 (6 h)	Qualitative motility	Fast	Fast	Fast	Fast	Fast	Fast
Day 3	% Survival	36	69	63	61	93	88
	Qualitative motility	Fast	Fast	Fast	Fast	Fast	Fast
Day 5	% Survival	30	76	58	73	94	93
	Qualitative motility	Medium	Medium	Medium	Medium	Medium	Medium

*PVP* Polyvinyl pyrolidone

^a^PVP was added to a final concentration of 4 mM

### L3 infectivity in ferrets

All worms recovered from both treatment groups were alive and found only in the heart and pulmonary arteries of the ferrets. Adults derived from FCmf were recovered at a higher rate and were generally larger, based on worm length and weight, than adults resulting from CPmf. Of the 25 L3s inoculated into ferrets, 69% of the FCmf L3s and 52% of the CPmf L3s were recovered as adults (Table 3). No significant effect due to treatment was detected (*P* ≥ 0.1801) for the total number of worms recovered. On Day 113, FCmf L3s resulted in larger average male and female adults than CPmf L3s (0.5 and 1.0 cm, respectively), and the length of females from FCmf averaged approximately 1.3 cm larger than those from CPmf on Day 133, while males were similar in length (Table 4). No significant effects due to treatment were detected (*P* ≥ 0.4563) for the lengths of males and females recovered on Day 133 (Table 5). Males and females derived from FCmf grew in length 21 and 34%, respectively, between Days 113 and 133; in comparison, males and females derived from CPmf grew 24 and 35%, respectively, during the same period. Adult males and females produced from FCmf were larger by weight (22 and 28%, respectively) on Day 133, and females were approximately 30% larger than males in both treatment groups. Treatment differences between T01 and T02 were not significant (*P* ≥ 0.0818) for the average weights of males, females or total worms recovered from each animal on Day 133 (Table 6).

**Table 3:** Adult *D. immitis* recovery counts from ferrets on Day 133 (and Day 113 as indicated) from microfilariae freshly collected from a dog or cryopreserved

Treatment^a^	Animal ID	Live worm counts			
		Males	Females	Total	% Recovery
T01—FCmf	C79	10	9	19	76
	C88	6	12	18	72
	C63^b^	7	8	15	60
	Mean worm counts	7.7	9.7	17.3	69.3
	SD	2.1	2.1	2.1	8.3
T02—CPmf	C61	8	10	18	72
	C64	6	5	11	44
	C83^b^	4	6	10	40
	Mean worm counts	6.0	7.0	13.0	52.0
	SD	2.0	2.6	4.4	17.4
	Percentage reduction of mean worm counts from T01			24.9%	
*P*-value T01:T02 (two-tailed Student’s *t*-test)				0.1801	

*SD* Standard deviation

^a^Animals allotted to T01 received L3s derived from mf freshly collected from a dog (FCmf); animals allotted to T02 received L3s derived from mf had been cryopreserved for 4 weeks (CPmf)

^b^Animals removed from the study on Day 113

**Table 4:** Adult *D. immitis* length of intact worms recovered from ferrets on Days 113 and 133

	Worm length (cm)^a^											
	T01—FCmf						T02—CPmf					
	C63^b^		C79		C88		C83^b^		C61		C64	
	Male	Female	Male	Female	Male	Female	Male	Female	Male	Female	Male	Female
	8	9.5	10	13.5	13	17.5	8.5	8.3	11	16	11.8	12.5
	8.5	10.5	11	14	11.1	13.5	7.5	10	10.1	13	11.4	13
	9	11.5	11	14.1	12.1	12.5	8.5	8.5	11.1	14.9	10	10.5
	8.5	9	11.1	13.8	11.8	17.1	8	9.2	11.6	13.7	11.4	13.7
	9.2	9.5	10.8	14.5	12	17.5			10.4	13.8	9.3	13.5
	8		9.7	13.1	12.6	17.6			10.6	15.2		
	9		9.9	12.5		16.2			11.1	14.1		
			9.5			17.7			9.8	14.2		
			9.2			16.4				13.4		
			10			14.3				14.8		
						17.1						
						14.5						
*n*	7	5	10	7	6	12	4	4	8	10	5	5
Mean	8.6	10.0	10.2	13.6	12.1	16.0	8.1	9.0	10.7	14.3	10.8	12.6
SD	0.49	1.00	0.70	0.67	0.66	1.81	0.48	0.77	0.60	0.91	1.07	1.28

^a^Worms that were intact after dissection from the heart were measured in centimeters and averaged for each animal

^b^Animals C63 (T01) and C83 (T02) were necropsied on Day 113. All other animals were necropsied on Day 133

**Table 5:** Statistical analysis of adult *D. immitis* length of worms recovered from ferrets on Day 133

Treatment	Animal ID	Average worm length (cm) on Day 133	
		Male	Female
T01—FCmf	C79	10.2	13.6
	C88	12.1	16
T02—CPmf	C61	10.7	14.3
	C64	10.8	12.6
*P*-value T01:T02 (two-tailed Student’s *t*-test)		0.7375	0.4563

Only worms that were intact following dissection were included

**Table 6:** Statistical analysis of adult *D. immitis* weight of worms recovered from ferrets

Treatment	Animal ID	Average worm weight (mg) on Day 133		
		Male	Female	Total
T01—FCmf	C79	24.8	42.7	32.2
	C88	22.0	33.3	29.6
T02—CPmf	C61	19.1	28.1	24.1
	C64	17.5	22.6	19.8
*P*-value T01:T02 (two-tailed Student’s *t*-test)		0.0818	0.1342	0.0843

Only intact worms recovered on Day 133 were included

### L3 infectivity and fitness in dogs

Bi-weekly sampling for mf from the dogs inoculated with L3s from CPmf and FCmf began at week 20 (Day 140). No dogs showed mf until Week 26 (Day 182), at which time two of the dogs inoculated with CPmf and one dog inoculated with FCmf began to show patent infections (Table 7). All four animals were antigen positive at 27 weeks (Day 198) post-inoculation. A second dog inoculated with FCmf was patent by Week 30 (Day 211). mf counts for all the dogs increased at a similar rate. No significant differences in mf production were detected between FCmf- and CPmf-inoculated dogs at any of the observed time points (*P* ≥ 0.3575). The dogs inoculated with L3s from CPmf were patent until necropsy. Approximately 50% of the inoculated L3s from FCmf were recovered as adults at 1.5 years post-inoculation (7 females and 7 males; in total 14 females and 14 females for the two animals). For CPmf, aproximately 60% of the inoculated L3s were recovered as adults at 2 years (18 females, 9 males) and 3.5 years (7 females, 16 males) post-inoculation.

**Table 7:** Biweekly microfilariae counts in dogs following inoculation with 40 infective L3 larvae

Treatment	Animal ID	Microfilariae counts at specific time points (weeks post-inoculation)				
		24	26	28	30	36
T01—FCmf	2540003	0	24	47	370	4000
	2420440	0	0	0	11	0
	Average	0	12.0	23.5	190.5	2000
T02—CPmf	2432880	0	7	54	220	10,000
	2418950	0	15	88	450	15,000
	Average	0	11.0	71.0	335.0	12,500
*P*-value T01:T02 (two-tailed Student’s *t*-test)		–	0.6690	0.3575	0.4686	0.3323

Counts were not collected for Week 32 or 34

## Discussion

Based on the results of our experiments, the survival of mf over a 5-day period in the six tested cryoprotectants/comination of cryoprotectants can be categorized into three groups: high, medium and low. Glycerol alone (5%) was clearly inferior to all of the other cryoprotectants tested, with survival rates of around 30% on Day 3 and Day 5 post-thaw. Medium survival rates were seen with DMSO alone, at 5 and 6%, respectively, and with 5% glycerol/4 mM PVP, with a survival rate of approximately 65% on Day 3 and between 58 and 76% on Day 5 post-thaw. High post-thaw survival rates (> 85% on Days 3 and 5) were seen with the combinations 5% DMSO/4 mM PVP and 6% DMSO/4 mM PVP. These results suggest that DMSO and PVP were both important contributors to the maximization of survival of frozen mf.

Previous reports from the ferret model for heartworm infection show infection rates of 100% and adult recoveries of between 34 and 54% [13]. The results our small ferret study showed 100% infection rates for CPmf and FCmf, and similar adult recovery (52 and 69% for CPmf and FCmf, respectively). In our study, cryopreservation appeared to have a negative impact on *D. immitis* development in ferrets: in comparison to FCmf worms, fewer CPmf worms survived to the adult stage following inoculation, and the surviving worms were smaller by weight and length. However, over the 20 days from Day 113 to Day 133 the growth rate for CPmf appears to be at least equal to, if not slightly higher than, that of FCmf. Although the sample size of the experiment is too small to draw definitive conclusions, these results suggest that the deleterious effects of cryopreservation may lessen as the worms mature in the host.

The most important aspect of these studies was to confirm that the *D. immitis* life-cycle could be completed following cryopreservation. Where the ferret study provided insights into the general health of the worms following a cryopreservation event, the dog study confirmed that adults from CPmf could produce viable mf. It has been reported that the onset of patency can begin as early as 6 months [1] and as late as 7–9 months [14, 15]. With the exception of one dog (ID 2420440; FCmf), the onset of patency in this study began at 26 weeks (5.5 months) following inoculation, and mf production was similar through 36 weeks for adults from both sources. Low numbers of mf counts were recorded for dog 2420440 on week 30, but not at week 36. This animal showed a positive adult heartworm antigen test at 27 weeks post-inoculation, indicating that adult worms were present. These results suggest that this dog may have been developing an immune-mediated occult infection, where mf are attacked by the host immune system and are eliminated from the circulatory system [16]. The CPmf dogs developed robust mf infections, and these results indicate that cryopreservation of *D. immitis* mf does not appear to have a qualitative or quantitative impact on reproductive fitness. Additional studies in larger numbers of dogs should be conducted to confirm these results and to statistically validate that cryopreservation does not negatively impact infectivity rates and subsequent worm recovery. Additional studies on worm response to preventative drug administration should also be considered to ensure these procedures do not impact true worm viability and fitness. Cryogenically preserved mf used for the ferret and dog studies were frozen for 4 and 12 weeks, respectively. This duration was deemed an appropriate length of time to assess survivabilty following short-term cryopreservation. Studies investigating longer durations of cryopreservation should be conducted to confirm how long these parasites can be maintained in the cryopreserved state without causing negative effects on infectivity.

The authors believe these studies confirm that cryopreservation methods may be used to maintain heartworm isolates/strains in a viable state. This offers a tremendous research advancement, not only in being able to preserve these parasites in a viable state for future use, but also to greatly reduce the requirement for canine hosts to maintain these parasites for research purposes.

## Conclusions

These are the first studies to establish that mf of *D. immitis* can be successfully cryopreserved, thawed and passaged through the mosquito vector to produce L3s. These L3s were infective to ferrets and dogs following artificial inoculation, and subsequently produced viable, reproductively fit adult worms in dogs, which in turn produced viable mf and completed the life-cycle. Mf produced from the original passage were infective upon a second passage through mosquitoes and dogs, validating further the quality of the cryopreservation procedures.

## Data Availability

All relevant data supporting the conclusions of this article are included within the article.

## Abbreviations

CPmf: Cryopreserved microfilariae
DMSO: Dimethyl sulfoxide
EDTA: Ethylenediaminetetraacetic acid
FCmf: Freshly collected microfilariae
HBSS: Hank’s Balanced Salt Solution
L3s: Third-stage larvae
mf: Microfilariae
ML: Macrocyclic lactones
PVP: Polyvinyl pyrolidone
